# Ephrin B2 and Ephrin B3 are receptors for a novel putative henipavirus with zoonotic potential

**DOI:** 10.1371/journal.pntd.0014557

**Published:** 2026-07-17

**Authors:** Guimin Dai, Shuang Yao, Wenjie Chen, Jinge Zhang, Xiaoyu Du, Yan Zhao, Zhenming Jin, Guigen Zhang

**Affiliations:** 1 Institute of Human Virology, Key Laboratory of Tropical Disease Control of Ministry of Education, Zhongshan School of Medicine, Sun Yat-sen University, Guangzhou, China; 2 Department of Otolaryngology Head and Neck Surgery, Beijing Tongren Hospital, Capital Medical University, Beijing, China; 3 Hunan Research Center of the Basic Discipline for Cell Signaling, College of Biology, Hunan University, Changsha, China; NIAID Integrated Research Facility, UNITED STATES OF AMERICA

## Abstract

Next-generation sequencing has accelerated the discovery of novel putative viruses in wildlife reservoirs, while identifying those with zoonotic potential remains challenging. In this study, we report the identification and characterization of Ailong virus, a novel putative henipavirus from previous bat metagenomes in China that utilizes human ephrin B2 (EFNB2) and EFNB3 as functional receptors. Using an integrated approach combining phylogenetic analysis, pseudotyped virus entry assays, antibody blockade assays, and structural modeling, we demonstrate that Ailong virus glycoprotein binds human EFNB2 and EFNB3 with high specificity, mediating pseudovirus entry into both human neuronal and respiratory epithelial cells. Structural analysis revealed the Ailong virus glycoprotein–EFNB2 interface closely resembling that of Nipah virus (NiV), with conservation of all critical receptor-binding residues. Moreover, AiV encodes an exceptionally large phosphoprotein, 1,033 amino acids in length, which is larger than any other known phosphoprotein in the subfamily *Paramyxoviridae*. Given its receptor usage, structural similarities to NiV, and efficient entry in human airway epithelia, Ailong virus is believed to pose a spillover risk.

## Author summary

Henipaviruses belong to the family *Paramyxoviridae*, a group of enveloped, nonsegmented, single-stranded RNA viruses. Among them, Nipah virus (NiV) and Hendra virus (HeV) are major zoonotic pathogens that cause encephalitis and respiratory infections in humans and animals. Nowadays, with the development of next-generation sequencing (NGS) and bioinformatics, huge amounts of putative viruses have been discovered in wildlife reservoirs. It is essential to identify the putative viruses with zoonotic potential and assess their spillover risks. From a previous metagenomics study, we identified a novel putative henipavirus, Ailong virus (AiV), which is phylogenetically close to NiV and HeV. Moreover, similar to NiV and HeV, AiV also utilizes human ephrin B2 (EFNB2) and ephrin B3 (EFNB3) as receptors. The Ailong virus glycoprotein–EFNB2 interface closely resembles that of NiV. Similar to NiV, AiV pseudovirus is able to efficiently enter human neuronal and respiratory epithelial cells, including differentiated human nasal epithelial cells (hNECs) under air-liquid interface (ALI) conditions. Our study suggests that AiV is a novel putative henipavirus with zoonotic potential.

## Introduction

Emerging infectious diseases of zoonotic origin account for approximately 75% of recent human pathogen introductions, highlighting the persistent threat of viral spillover events from animal reservoirs [1]. The advent of next-generation sequencing (NGS) and bioinformatics has revolutionized viral discovery, enabling the identification of novel viruses through metagenomics. Recent advances in AI-driven strategies have further accelerated this process, with over 160,000 previously unknown viruses detected through machine learning algorithms [2]. Virus discovery is of great importance in filling gaps in the evolutionary history of viruses and improving our understanding towards the animal origins of novel zoonoses [3]. Predicting which virus from the metagenomic sequences might infect humans is challenging. In principle, applying designed experiments to these newly discovered viruses provides valuable information. However, such experiments remain laborious and cannot keep pace with the dramatic acceleration of virus discovery [4]. Thereby, the majority of viruses are not assessed for their zoonotic spillover risks.

Hendra virus (HeV) and Nipah virus (NiV), two prototype henipaviruses, were first reported in Australia in 1994 and in Malaysia in 1998–1999, respectively. Both cause severe encephalitis and acute respiratory infections in humans and animals [5,6]. Despite being designated a WHO priority pathogen, no approved vaccines or antivirals for NiV exist. Recurrent outbreaks of NiV have occurred in India and Bangladesh since 2001 [7,8]. Both NiV and HeV utilize ephrin B2 (EFNB2) as their primary entry receptor, a highly conserved protein across mammalian species that serves as a key determinant of cross-species transmission potential [9,10]. Besides, NiV can also use ephrin B3 (EFNB3) as an alternative receptor [11,12].

Recent metagenomic surveys have identified numerous novel putative henipa-like viruses or viral sequences in animal reservoirs, yet their capacity to infect human cells remains uncharacterized [13]. We hypothesized that conservation of critical EFNB2-binding residues in viral glycoproteins could predict zoonotic potential without requiring high-containment viral culture. This approach is also necessary as there is no viral isolate. Through phylogenetic and structural analysis of metagenomically-derived henipa-like virus glycoprotein sequences, we identified a novel putative virus—designated Ailong virus (AiV)—that maintains these conserved residues critical for human EFNB2 binding. Here, we comprehensively characterize AiV pseudovirus receptor usage, demonstrate its efficient entry in human respiratory and neuronal cells, and elucidate the structural basis of its interaction with human EFNB2. Our findings reveal AiV as an uncharacterized pathogen with high zoonotic risk.

## Results

### Identification of a novel putative EFNB2-binding henipavirus in Chinese bat metagenomes

**Fig 1:**
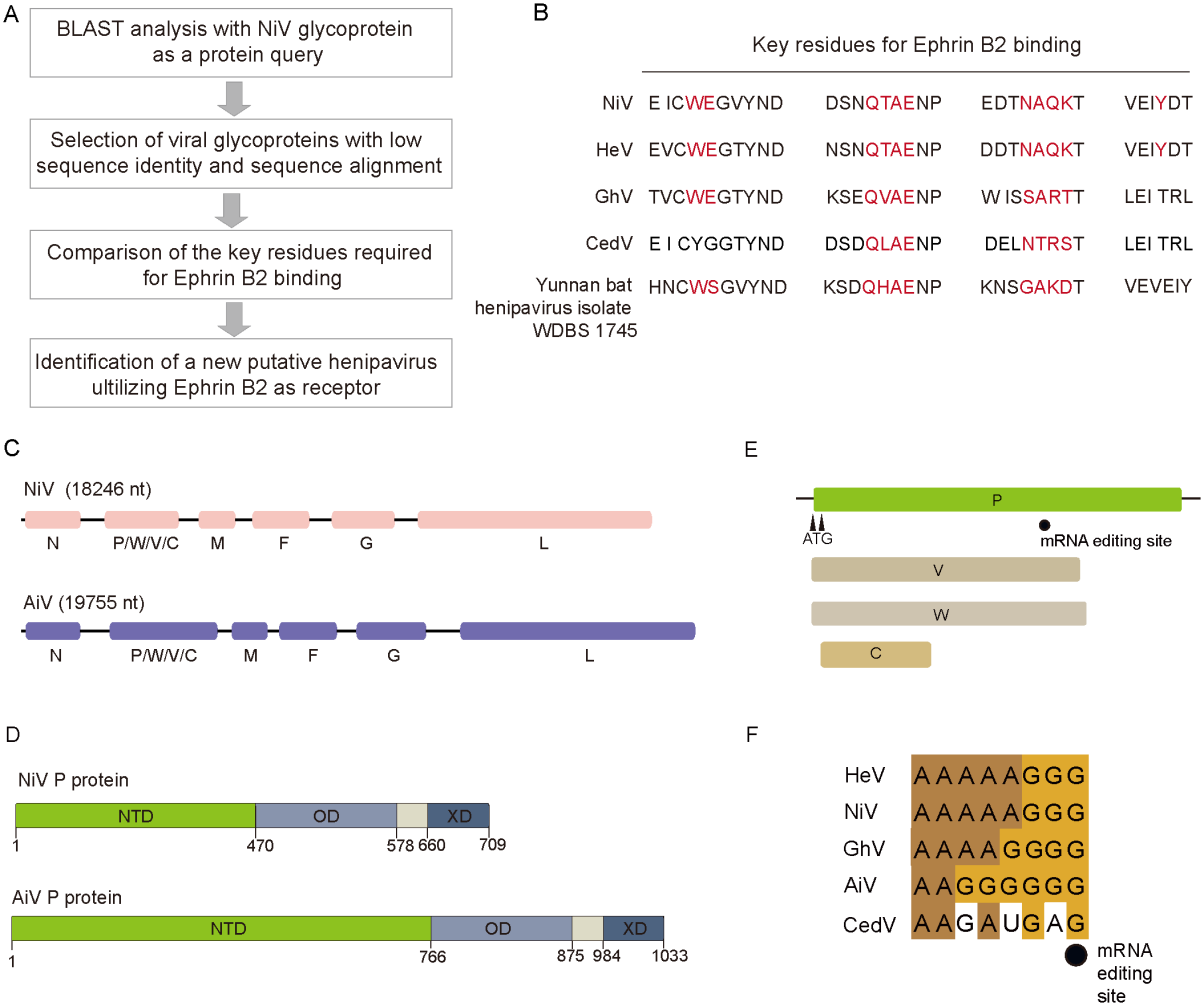
Identification of a viral glycoprotein with conserved residues critical for EFNB2 binding from Chinese bat metagenomes. **(A)** Flowchart for identification of viral glycoproteins that bind to human ephrin B2. **(B)** Sequence analysis of key residues in glycoprotein proteins from NiV (AAK29088.1), HeV (AAC83193.2), Ghana virus (GhV, YP_009091838.1), Cedar virus (CedV, YP_009094086.1) and the new henipavirus (XRT74647.1) that interact with the receptor ephrin B2. The key residues are highlighted in red. **(C)** The genome size and organization of AiV and NiV. The coding regions for each gene are shown and depicted in color. Noncoding intergenic regions are indicated by black lines. **(D)** Schematic diagram of the phosphoproteins (P) of NiV (AAF73378.1) and AiV (XRT74644.1). Colored bars: N-terminal domain (NTD, brown), oligomerization domain (OD, gray), and C-terminal X domain (XD, blue). **(E)** Schematic of the P gene of AiV. The alternative translation start sites are indicated by black triangle. The putative mRNA editing site is indicated by solid circle. **(F)** Sequence alignment of the putative mRNA editing site across members of the genus *Henipavirus*.

To systematically evaluate zoonotic potential among metagenomically-derived henipaviruses, we analyzed glycoprotein sequences from over 85 virus candidates using NiV glycoprotein as a reference (Fig 1A). Despite overall low sequence identity (<30%) across most candidates, we focused on conserved residues critical for EFNB2 binding with the glycoproteins. The residues (W504, E505, Q530, T531, A532, E533, N557, A558, Q559, Y581) in NiV glycoprotein were previously validated as essential for EFNB2 binding [9–11,14,15]. This approach identified a single candidate, Yunnan bat henipavirus isolate WDBS1745 [16], which contains these conserved residues, indicating its EFNB2-binding capability (Fig 1B). Phylogenetic reconstruction based on nucleocapsid protein (N) sequences and complete genome sequences demonstrated that WDBS1745 clusters with NiV and HeV, occupying a basal position within the *Henipavirus* genus (S1 Fig). Subsequent genomic analysis revealed additional distinctive features, most notably an exceptionally large phosphoprotein (1,033 amino acids, aa), which is larger than any other known phosphoprotein in the subfamily *Paramyxoviridae* (Figs 1C and 1D). Comparatively, the P proteins of NiV, HeV and Cedar virus (CedV) are 709, 707, and 737 aa in length, respectively. Based on its unique phylogenetic position, receptor-binding characteristics, and genomic architecture, we propose classification of this isolate WDBS1745 as Ailong virus (AiV), a putative EFNB2-utilizing henipavirus. We also compared the sequences of AiV-encoded proteins, including nucleoprotein (N), phosphoprotein (P), C protein, matrix protein (M), fusion protein (F), attachment glycoprotein (G), and the large protein or RNA polymerase protein (L), with those of NiV, HeV, and CedV, respectively (S1 Table). Among them, the nucleoprotein (N) is relatively conserved, which is approx. 70% identical to that of NiV and HeV (S1 Table). Similar to NiV, a highly conserved RNA editing site is present in the phosphoprotein (P) gene of AiV (Figs 1E and 1F).

### Human EFNB2 and EFNB3 serve as the functional receptors for AiV entry

**Fig 2:**
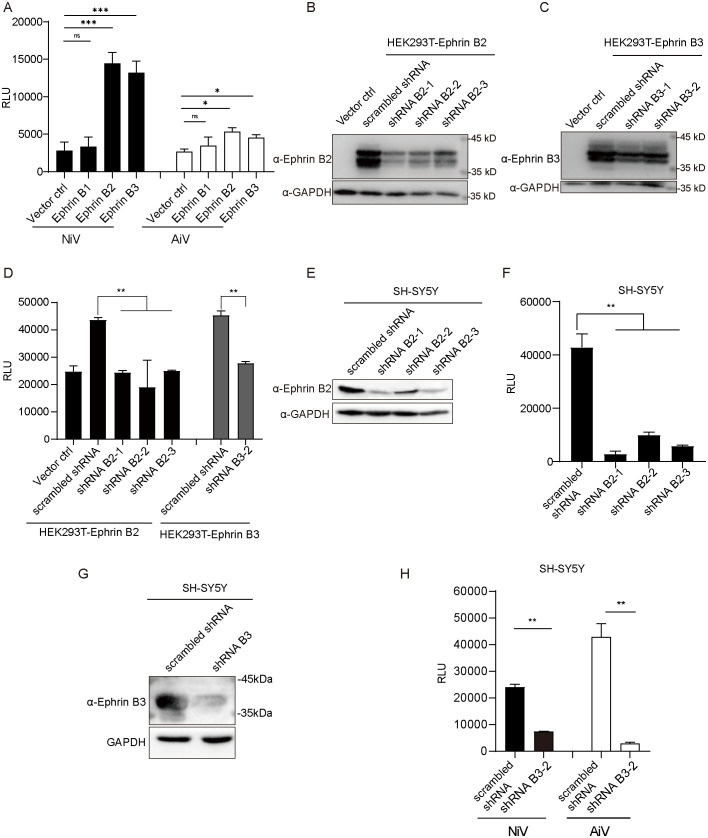
AiV utilizes ephrin B2 and ephrin B3 as functional receptors. **(A)** HEK293T cells overexpressing EFNB1, EFNB2, EFNB3, or a control vector were transduced with pseudotyped AiV or NiV particles. This experiment was performed in triplicate as independent biological replicates. **(B-C)** HEK293T cells overexpressing EFNB2 (B) or EFNB3 (C) were transfected with the corresponding *EFNB2, EFNB3 shRNA* or scrambled shRNAs. Knockdown efficiency was confirmed by Western blotting. These experiments were conducted in duplicate as independent biological replicates. **(D)** *EFNB2*, *EFNB3* knockdown or control cells were transduced with AiV pseudoviruses and the luciferase activity was measured. This experiment was performed in triplicate as independent biological replicates. **(E-F)** SH-SY5Y cells were transduced with lentiviral vectors expressing *EFNB2* or scrambled shRNAs. EFNB2 knockdown was confirmed by Western blotting **(E)**. The knockdown and control cells were transduced with AiV pseudoviruses **(F)**. **(G-H)** Similarly, SH-SY5Y cells were transduced with lentiviral vectors expressing EFNB3 or scrambled shRNAs. The knockdown efficiency of EFNB3 was confirmed by Western blotting **(G)**. The knockdown and control cells were transduced with AiV or NiV pseudoviruses **(H)**. Statistical significance was determined by two-tailed Student’s t-test (ns, not significant; *p < 0.05, **p < 0.01, ***p < 0.001).

**Fig 3:**
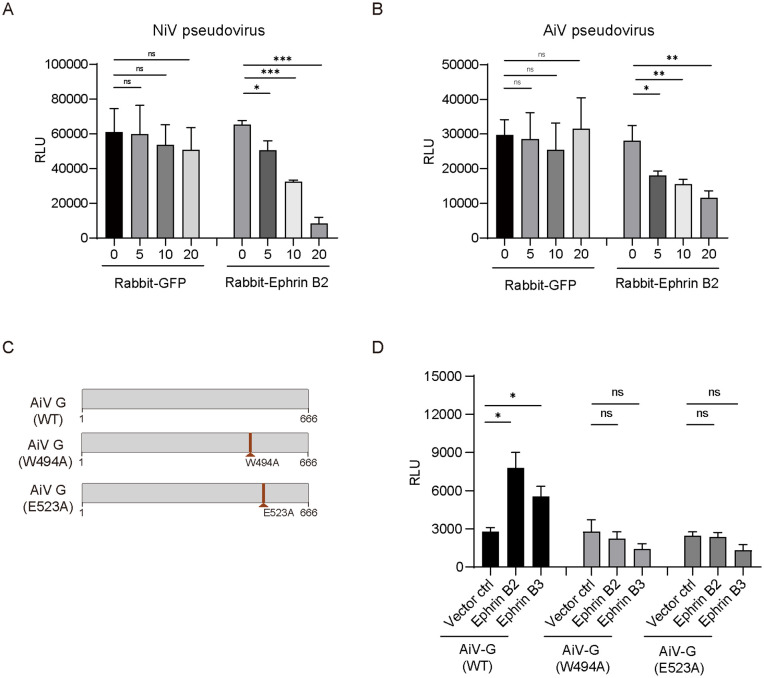
Ephrin B2 antibody treatment inhibited AiV pseudovirus cell entry. **(A-B)** Huh7 cells were pre-incubated with GFP antibody or Ephrin B2 antibody at the indicated concentrations for 2 hours, then transduced with NiV (A) or AiV (B) pseudoviruses. Luciferase activity was measured 48 hours after transduction. **(C)** Schematic diagram of AiV G mutants. The mutation sites are highlighted in red. **(D)** HEK293T cells overexpressing EFNB2, EFNB3, or a control vector were transduced with AiV pseudoviruses bearing AiV G (WT), W494A or E523A mutant. Statistical significance was determined by two-tailed Student’s t-test (ns, not significant; *p < 0.05, **p < 0.01, ***p < 0.001).

To validate the receptor usage of Ailong virus (AiV), we conducted both overexpression and knockdown experiments in different human cells. First, human EFNB1, EFNB2, or EFNB3 was overexpressed in HEK293T cells. Pseudotyped lentiviral particles containing AiV glycoprotein (G) and fusion protein (F) were generated. Pseudotyped lentiviral particles bearing NiV-G and F proteins were used as positive control. As shown in Fig 2A, both NiV and AiV pseudoviruses exhibited efficient entry into EFNB2- or EFNB3-overexpressing HEK293T cells, confirming the functional requirement of EFNB2 or EFNB3 for viral entry. In comparison with EFNB2, EFNB3 showed lower efficiency in mediating AiV pseudovirus entry (Fig 2A). To further interrogate the receptor dependency, we silenced *EFNB2 or EFNB3* expression with shRNAs in HEK293T cells expressing *EFNB2* or *EFNB3* (Fig 2B and 2C). As shown in Fig 2D, knockdown of *EFNB2 or EFNB3* reduced AiV cell entry. Similar dependency was observed in human neuroblastoma SH-SY5Y cells, a neuronal model permissive to henipavirus infection. Endogenous *EFNB2* or *EFNB3* knockdown in SH-SY5Y cells resulted in significant reduction in AiV pseudovirus entry (Fig 2E and 2H). To further confirm the usage of EFNB2 as a receptor of AiV, we treated Huh7 cells with the EFNB2-specific antibody. As shown in Fig 3A and 3B, blockade of EFNB2 with specific antibody inhibited both NiV and AiV pseudoviruses cell entry. The residues W494 and E523 of AiV glycoprotein are highly conserved in AiV, NiV and HeV, and are essential for EFNB2 binding. We therefore generated two AiV glycoprotein (G) mutants, W494A and E523A (Fig 3C). As shown in Fig 3D, AiV pseudovirus bearing W494A or E523A mutant was unable to enter target cells even in the presence of EFNB2 or EFNB3. Collectively, these results confirm the requirement and specificity of EFNB2 and EFNB3 for AiV entry.

### AiV glycoprotein demonstrates interaction with EFNB2 and EFNB3

**Fig 4:**
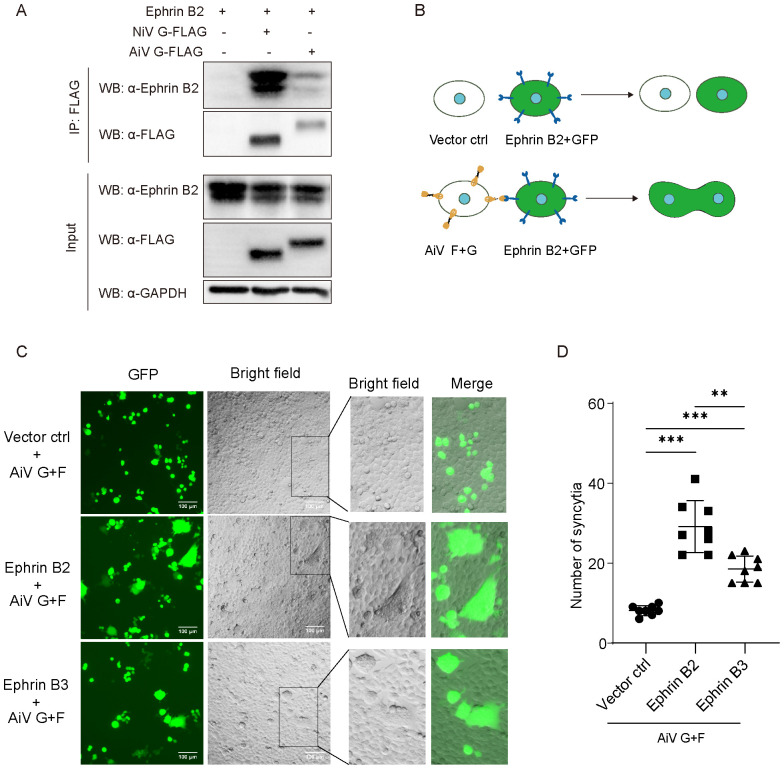
The glycoprotein of AiV binds to human ephrin B2 and ephrin B3. **(A)** Coimmunoprecipitation of AiV glycoprotein and ephrin B2. HEK293T cells were co-transfected with EFNB2 and FLAG tagged NiV or AiV glycoprotein (G) expression vector. Forty-eight hours after transfection, the cell lysates were subjected to immunoprecipitation using anti-FLAG magnetic beads, followed by Western blotting with the indicated antibodies. **(B)** Schematic diagram of AiV glycoprotein and fusion protein-mediated syncytia formation. **(C)** HEK293T cells transfected with AiV glycoprotein and fusion protein were co-cultured with HEK293T cells expressing GFP and EFNB2, EFNB3, or the vector control. The cells were imaged via fluorescence microscopy to visualize the syncytia. Scale bar = 100 μm. **(D)** Quantification of syncytia from eight randomly selected microscopic fields per group. The data are representative of three independent experiments. Statistical significance was determined by two-tailed Student’s t test, where ***p < 0.001.

We next performed co-immunoprecipitation (co-IP) assays in HEK293T cells co-expressing FLAG-tagged AiV glycoprotein (AiV-G) or NiV glycoprotein (NiV-G) with EFNB2. The NiV-G was used as a positive control here. As shown in Fig 4A, EFNB2 specifically co-precipitated with NiV-G, as well as AiV-G, though the precipitated EFNB2 together with AiV-G was less than that with NiV-G. These results confirm the protein-protein interaction between AiV-G and EFNB2. When binding its receptor, AiV glycoprotein (AiV-G) can induce syncytia in the presence of fusion protein (AiV-F). To assess functional receptor engagement, we evaluated syncytia formation in HEK293T cells co-expressing AiV-G, AiV-F with EFNB2 or EFNB3 (Fig 4B). As demonstrated in Fig 4C-4D, the glycoprotein (G) and fusion protein (F) of AiV efficiently induced syncytia formation in the presence of EFNB2 or EFNB3, while the glycoprotein (G) or fusion protein (F) alone was not able to induce syncytia formation (S2 Fig). These results further confirm that the glycoprotein of AiV binds to human EFNB2 and EFNB3.

### Structural basis of AiV glycoprotein-receptor interaction

**Fig 5:**
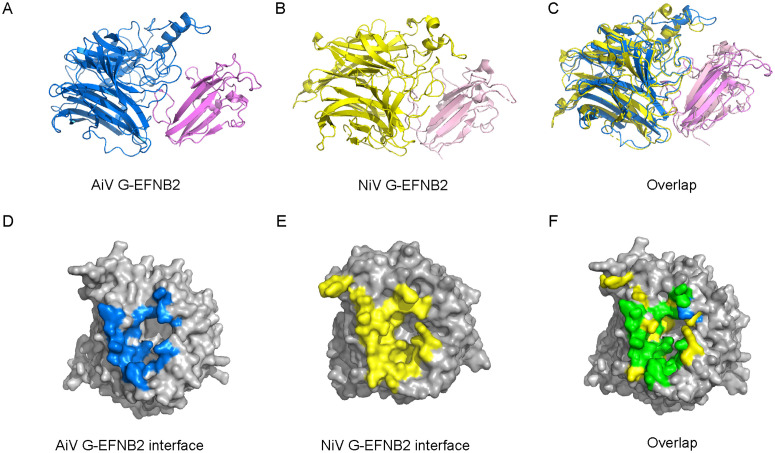
Evolutionarily conserved EFNB2 recognition architecture in henipaviral glycoproteins. **(A)** AiV G (blue)–EFNB2 (purple) complex structure predicted by AlphaFold3. **(B)** Experimental NiV G (yellow)–EFNB2 (pink) complex structure (PDB: 2VSM). **(C)** Structural superposition of AiV G-EFNB2 complex and NiV G-EFNB2 complex revealing conserved binding topology. **(D,E)** Receptor-binding interfaces of AiV (blue) and NiV (yellow), highlighting conserved motif positions. **(F)** Superimposed interfaces (green) demonstrate spatial overlap of core receptor-contact residues across both viruses.

We further determined the structure of AiV-G in complex with human EFNB2 using AlphaFold3 (Fig 5A), achieving a predicted template modeling (pTM) score of 0.80. The previously resolved crystal structure of NiV-G/EFNB2 complex (PDB: 2VSM) served as a reference architecture here (Fig 5B). Structural superposition revealed a conserved binding mode with a backbone root mean square deviation (RMSD) of 1.886 Å (Fig 5C), confirming the evolutionary conservation of receptor-binding architecture between AiV-G and NiV-G. The AiV-G/EFNB2 interface spans 916 Å², containing ten hydrogen bonds and extensive hydrophobic interactions (Fig 5D and S3 Fig). Notably, all the critical receptor-binding residues identified in NiV were structurally conserved in AiV, forming identical spatial arrangements despite only 28% overall sequence identity between the viral glycoproteins (Fig 5D and 5E). Structural clustering analysis demonstrated that the overlapping interface region adopts a near-identical conformation (RMSD = 0.39 Å), containing all critical residues for EFNB2 recognition (Fig 5F). This structural conservation suggests both viruses maintain receptor-binding specificity despite divergent evolutionary trajectories.

### AiV pseudovirus efficiently enters differentiated human nasal epithelial cells

**Fig 6:**
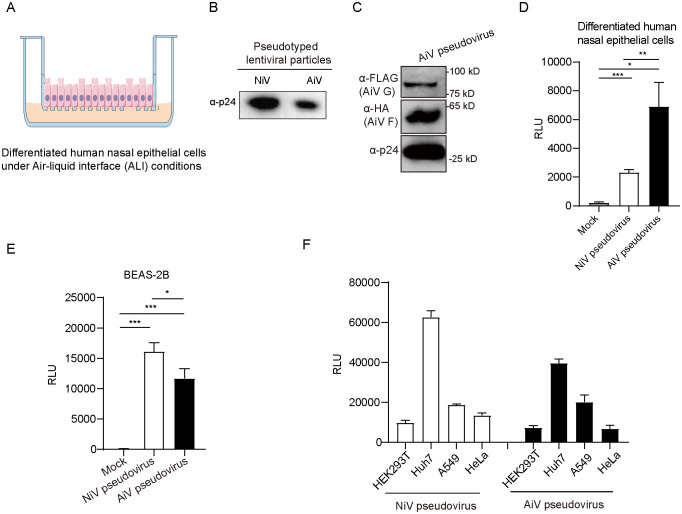
AiV pseudovirus is able to enter differentiated human nasal epithelial cells. **(A)** Schematic of differentiated human nasal epithelial cells under air-liquid interface (ALI) conditions. **(B)** The amount of pseudotyped lentiviral particles was quantified by Western blotting with anti-p24 antibody. **(C)** The incorporation of HA-tagged F and FLAG-tagged G proteins into the AiV pseudoviruses was verified by Western blotting. **(D)** Differentiated human nasal epithelial cells were transduced with pseudotyped NiV or AiV particles. After incubation with the pseudoviruses for 4 h, cells were maintained in ALI differentiation medium for 48 h and subsequently lysed for luciferase measurement. This experiment was conducted with two biological replicates. **(E-F)** BEAS-2B, HEK293T, Huh7, A549, and HeLa cells were transduced with pseudotyped NiV or AiV. Luciferase activity was measured 48 h post-transduction. The experiments in (E) and (F) were conducted in two and three biological replicates, respectively. Statistical significance was determined by two-tailed Student’s t-test, where *p < 0.05, **p < 0.01, ***p < 0.001.

To assess physiological relevance, we established differentiated human nasal epithelial cells (hNECs) under air-liquid interface (ALI) conditions (Fig 6A), a validated model for human mucosal barrier studies [17]. Pseudotyped lentiviral particles bearing AiV or NiV glycoproteins (G) were generated and used for virus entry assays. The loads of pseudotyped lentiviral particles to hNECs were quantified by measurement of the p24 protein with Western blotting (Fig 6B). The incorporation of AiV G and F into the pseudotyped lentiviral particles was also confirmed by Western blotting with specific antibodies (Fig 6C). As shown in Fig 6D, both pseudotyped viruses exhibited efficient entry into hNECs. Further, we tested the permissiveness of immortalized human lung epithelial cells BEAS-2B. Indeed, BEAS-2B cells were susceptible to both AiV and NiV, too (Fig 6E). We also performed the pseudovirus-based entry assays in other human cells, including HEK293T, Huh7, A549 and HeLa cells. Consistently, AiV and NiV showed comparable cell tropism and entry efficiency in these cells (Fig 6F). These results indicate that AiV is human-infecting and poses a significant threat to global health.

## Discussion

The discovery of Ailong virus as the world’s fifth putative henipavirus—and the first in China—with EFNB2 and EFNB3 as functional receptors, represents a significant advance in our understanding of henipavirus diversity and zoonotic potential. The recent ICTV recognition of *Parahenipavirus* underscores the evolutionary bifurcation between henipa-like viruses from rodent/shrew reservoirs and bat-borne lineages [18]. AiV belongs to the *Henipavirus* genus, as it is bat-borne. AiV’s classification within the *Henipavirus* genus is also supported by multiple lines of evidence: phylogenetic position based on conserved structural proteins, high conservation of EFNB2-binding residues, genome structure similarity to other henipaviruses, and potential capacity to infect human cells. The recently identified rodent-borne parahenipaviruses in China, Langya virus (LayV) and Mojiang virus (MojV), are grouped into the genus *Parahenipavirus*, which utilize different cellular receptors [19,20].

The exceptional length of AiV phosphoprotein represents a unique feature. The canonical RNA editing site is conserved in the phosphoprotein (P) gene, which enables production of V and W accessory proteins [21–23]. The extended N-terminal domain of AiV phosphoprotein may confer novel host interaction capabilities, which harbors intrinsically disordered regions (IDRs). Further studies are needed to determine whether this extension contributes to immune evasion or modulates viral polymerase activity.

Critically, AiV pseudovirus efficient entry in human nasal epithelia under air-liquid interface conditions demonstrates its potential human infectivity. AiV pseudovirus shows comparable entry efficiency with NiV in different human cells, suggesting AiV poses a high zoonotic risk. Though the pseudovirus-based entry assays provide evidence for EFNB2 and EFNB3-dependent entry, studies with authentic AiV under high biosafety containment are essential to fully evaluate its infectivity and transmission in animals. Furthermore, serological surveys of bat-exposed local human populations could reveal evidence of prior spillover events. The identification of AiV—the fifth putative EFNB2-utilizing henipavirus in the world and the first one in China—highlights the need for development of broad-spectrum vaccines and antiviral drugs against different henipaviruses. By integrating computational prediction with targeted experimental validation, as demonstrated here, we can prioritize the most concerning viral threats before they emerge as human pathogens.

## Materials and methods

### Cell culture

HEK293T, A549, Huh7, HeLa, SH-SY5Y, and BEAS-2B were obtained from the Cell Bank of Shanghai Institute of Biological Sciences, Chinese Academy of Sciences. The cells were cultured in Dulbecco’s Modified Eagle Medium (DMEM) supplemented with 10% fetal bovine serum (FBS) and 1% penicillin-streptomycin solution in an incubator at 37°C.

### Plasmid construction

The expression vectors of AiV glycoprotein (G) and fusion protein (F), pcDNA3.1-AiV-G-FLAG (GenBank: XRT74647.1) and pcDNA3.1-AiV-F-HA (XRT74646.1), were codon-optimized and synthesized (Genewiz, Suzhou, China). The expression vectors of NiV glycoprotein (Cat: VG40980-UT) and fusion protein (Cat: VG40985-CH) were purchased from Sino Biological, China. The FLAG tagged expression vector pcDNA3.1-NiV-G-FLAG was generated via subcloning. The expression vectors of human ephrin B2 (EFNB2) and ephrin B3 (EFNB3) were purchased from Sino Biological, China (Cat: MG50598-CY; Cat:HG20190-NF). Human ephrin B1 (EFNB1) was obtained from Bio-research innovation Center Suzhou. The HA tagged expression vector pcDNA3.1-ephrin B1-HA was generated via subcloning. The sequences of these vectors were verified by Sanger sequencing.

### BLAST analysis

To perform a BLAST analysis (https://blast.ncbi.nlm.nih.gov/Blast.cgi), the glycoprotein of NiV (QBQ56723.1) was chosen as a query. The database ClusteredNR (nr cluster seg) and Algorithm blastp (protein-protein BLAST) were used. The general parameters and scoring parameters were set as default values. Totally 86 viral glycoprotein sequences with low sequence identity were analyzed.

### Phylogenetic analyses

Phylogenetic trees were constructed based on the nucleocapsid (N) sequences of paramyxoviruses retrieved from GenBank. GenBank accession numbers are listed below. Denwin virus, OK623354.1; Angavokely henipavirus, ON613535.1; Cedar virus, JQ001776.1; Yunnan bat henipavirus isolate WDS1745, PQ621839.1; Yunnan bat henipavirus isolate WDS1733, PQ612840.1; Hendra virus, AF017149.1; Nipah virus (Malaysian), AJ627196.1; Nipah virus (Bangladesh), AY988601.1; Wufeng Chodsigoa smithii henipavirus 1, OM030316.1; Jingmen Crocidura shantungensis henipavirus 1, OM030314.1; Gamak virus, MZ574407.1; Daeryong virus, MZ574409.1; Melian virus, OK623353.1; Wenzhou shrew henipavirus 1, OQ715593.1; Mojiang virus, NC_025352.1; Langya virus, OM101125.1.

The sequences were aligned with ClustalW (default settings) and the phylogenetic tree was built via an online tool MEGA11 (v11.0.13 version). The neighbor-joining method with 1,000 bootstrap replicates was chosen. Branches with bootstrap values ≥70% were considered statistically significant.

### Production of pseudotyped lentiviral particles

HEK293T cells were co-transfected with vectors for glycoprotein, fusion protein, psPAX2, and pHIV-Luciferase at a 1:1:4:4 ratio. The pseudotyped lentiviral particles were collected and centrifuged at 6,000 × g for 10 min at 4°C to remove cellular debris. The supernatant was filtered through a 0.45 μm membrane filter (Millipore, USA). The production of pseudotyped lentiviral particles was verified by detection of p24 protein via Western blotting.

### Pseudovirus transduction and luciferase reporter assay

HEK293T cells were transfected with EFNB2, EFNB3, EFNB1 expression vector or a vector control using Lipofectamine 3000 (Thermo Fisher Scientific, USA). After 4–6 h incubation, the cells were re-seeded in fresh plates and incubated with pseudoviral particles for 5–6 h. Forty-eight hours after transduction, the cells were lysed in Passive Lysis Buffer (Promega, USA). The luciferase activity was measured using Luciferase Assay System (Promega, USA) with a GloMax 20/20 Luminometer (Promega, USA).

### Culture of human nasal epithelial cells under air-liquid interface (ALI) conditions

Nasal mucosal tissues from the inferior turbinate or uncinate process were obtained from patients undergoing septoplasty. This study was approved by the Ethics Committee of Beijing Tongren Hospital, and written informed consent was obtained from the participants prior to their participation. The tissues were placed in DMEM/F12 medium (Cytiva, USA) containing 0.05% proteinase K (Sigma, USA) and digested at 4 °C for 16–18 h. The digestion was terminated by adding fetal bovine serum (Cytiva, USA) to a final concentration of 10%. After vortexing for 20 s, the cell suspension was centrifuged at 1,000 rpm for 5 min at 4 °C. The supernatant was discarded, and the cells were resuspended in 1 ml DMEM/F12 medium. The suspension was then transferred to a 35 mm culture dish and incubated at 37 °C with 5% CO_2_ for 45 min allowing fibroblasts to attach and be removed.

The supernatant containing epithelial cells was then collected and centrifuged again at 1,000 rpm for 5 min at 4 °C. The collected cell pellet was resuspended in Bronchial Epithelial Cell Medium (BEpiCM; USA) and counted. Cells were seeded onto polyester membrane inserts (Corning, USA) in 6.5 mm-diameter Transwell chambers (Corning, USA) with a 0.4 μm pore size at a density of 1 × 10^5^ cells per well. The cells were then maintained at 37 °C in a 5% CO_2_ incubator, and the medium was changed every other day.

When the cells reached 80%-90% confluence, the medium in the Transwell chambers was removed, and the BEpiCM medium was replaced with an air-liquid interface (ALI) medium (DMEM/F12: BEpiCM = 1:1) to initiate epithelial differentiation under air-liquid interface conditions. The cultures were maintained at 37 °C in 5% CO_2_ with medium changes every other day. Transepithelial electrical resistance (TEER; unit: Ω × cm²) was measured using a Millipore Millicell ERS-2 voltohmmeter (Millipore, USA).

Upon stabilization of TEER values, the fully differentiated human nasal epithelial cells were transduced with NiV or AiV pseudoviruses, with three technical replicates for each group. Following 4 h incubation, the pseudoviral particles were removed and the cells were maintained in ALI differentiation medium for 48 h before lysis for luciferase activity measurement.

### Western blotting

The following antibodies were used in Western blotting: EFNB2 (ProteinTech, 1:2000 dilution), EFNB3 (Santa Cruz, 1:2000 dilution), GAPDH (ProteinTech, 1:10000 dilution), p24 (ProteinTech, 1:3000 dilution), FLAG (ProteinTech, 1:5000 dilution), HRP-conjugated goat anti-mouse or rabbit IgG secondary antibody (ProteinTech, 1:5000 dilution).

### shRNA Knockdown

HEK293T cells were co-transfected with pMD2.G, psPAX2, and the plasmid pLKO.1-shRNA to generate pseudotyped lentiviral particles. The following lentiviral vectors were constructed: (1) pLKO.1-EFNB2-shRNA, (shRNA- EFNB2–1: 5′-GCAGACAGATGCACAATTA-3′; shRNA- EFNB2–2: 5′-GAGACAAATTGGATATTAT-3′; shRNA- EFNB2–3: 5′-CGACAACAAGTCCCTTTGTAA-3′); (2) pLKO.1-EFNB3-shRNA, (shRNA- EFNB3–1: 5′-GCACCACGATTACTACATCAT-3′; shRNA- EFNB3–2: 5′-GTTCCAGGAGTATAGCCCTAA-3′; (3) pLKO.1-scrambled shRNA, a vector carrying a scrambled non-specific shRNA sequence, which was used as control. For vector construction, complementary forward and reverse oligonucleotides (Sangon Biotech, China) were synthesized and annealed to form double-stranded DNA inserts containing restriction enzyme sites (AgeI/EcoRI) and a hairpin loop structure (TTCAAGAGA). The knockdown efficiency was verified by Western blotting.

### Syncytia formation assay

One part of HEK293T cells were co-transfected with 1 μg of AiV glycoprotein (G) and 1 μg of fusion protein (F) expression vectors or control vector. A second of HEK293T cells were co-transfected with 1 μg GFP expression vector with 1 μg of EFNB2 or EFNB3 expression vector. Six hours post-transfection, the HEK293T cells were mixed together at a 1:1 ratio. Fluorescence images were acquired 48 h post transfection using an inverted fluorescence microscope (Leica DMI8, Germany, 20× objective).

### Structural modeling and interaction interface analysis

Computational prediction of the AiV glycoprotein (residues 176–595)–EFNB2 (residues 31–168) complex was performed using AlphaFold3 with default parameters. The top-ranked model (pLDDT >90 for interface residues) was selected for downstream analyses based on per-residue confidence metrics. Structural conservation relative to the NiV G–EFNB2 complex (PDB: 2VSM) was quantified through backbone superposition in PyMOL. Global similarity was assessed via iterative pairwise alignment (RMSD calculation over Cα atoms), with the align command applying default dynamic programming parameters. Interface residues were defined as pairs with interatomic distances ≤4.0 Å across binding partners. Spatial conservation of the receptor-binding pocket was evaluated by superimposing the interface regions of AiV and NiV complexes after global alignment. Visual validation of structural congruence and interface topology was performed prior to quantitative assessment.

## Supporting information

S1 Table Length and pairwise sequence identity of the predicted open reading frames of AiV and other henipaviruses. (TIF)

S1 Fig Phylogenetic analysis of the identified virus. The phylogenetic tree was constructed based on the nucleocapsid protein sequences (A) and complete genome sequences (B) of selected paramyxoviruses.
(TIF)

S2 Fig AiV G or F protein alone does not sufficiently induce syncytia formation. HEK293T cells transfected with AiV G (A and B), or F (C and D) were co-cultured with HEK293T cells expressing GFP together with EFNB2 or EFNB3, or the vector control. Cells were fixed and imaged by fluorescence microscopy to visualize the syncytia. Scale bar = 100 μm. (B, D) Quantification of syncytia from eight randomly selected microscopic fields per group. The experiments were conducted with three biological replicates. Data were analyzed by Student’s t-test (ns, not significant).
(TIF)

S3 Fig Intermolecular interactions at the AiV G–ephrin B2 interface. Hydrogen bonds are shown as green dashes; hydrophobic contacts as red/pink spoked arcs anchored to protein residues and directed toward ligand atom. The letter A in parentheses after an amino acid denotes AiV, and B in parentheses denotes EFNB2.
(TIF)

S1 File The Excel file (Origin values for graphs.xlsx) contains the origin data (values) for building graphs in different figures. (XLSX)

S2 File The archive file (The points extracted from images for Figures.rar) contains the origin images from which the points are extracted for analysis in figures 4D, S2A and S2B. (RAR)

S1 Raw Gel The archive file (Origin images for all blots.rar) contains the original images for all the blots. (RAR)
