# Supplementary figures and images for: Ephrin B2 and Ephrin B3 are receptors for a novel putative henipavirus with zoonotic potential
Source: PLoS Negl Trop Dis. 2026 Jul 17;20(7):e0014557. doi: 10.1371/journal.pntd.0014557 (PMC13421762; doi:10.1371/journal.pntd.0014557)

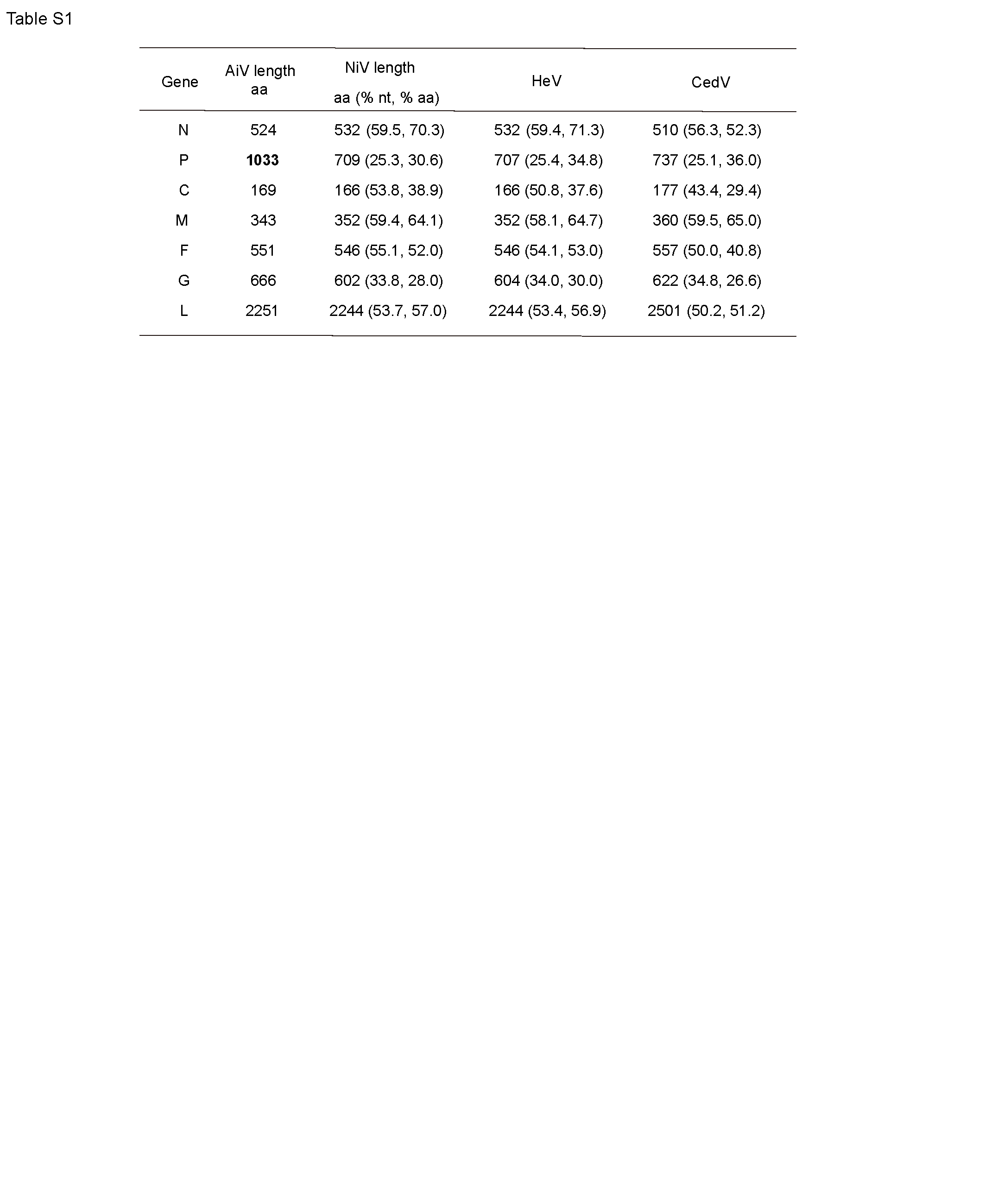

Supplement: S1 Table — (TIF) [file pntd.0014557.s001.tif]

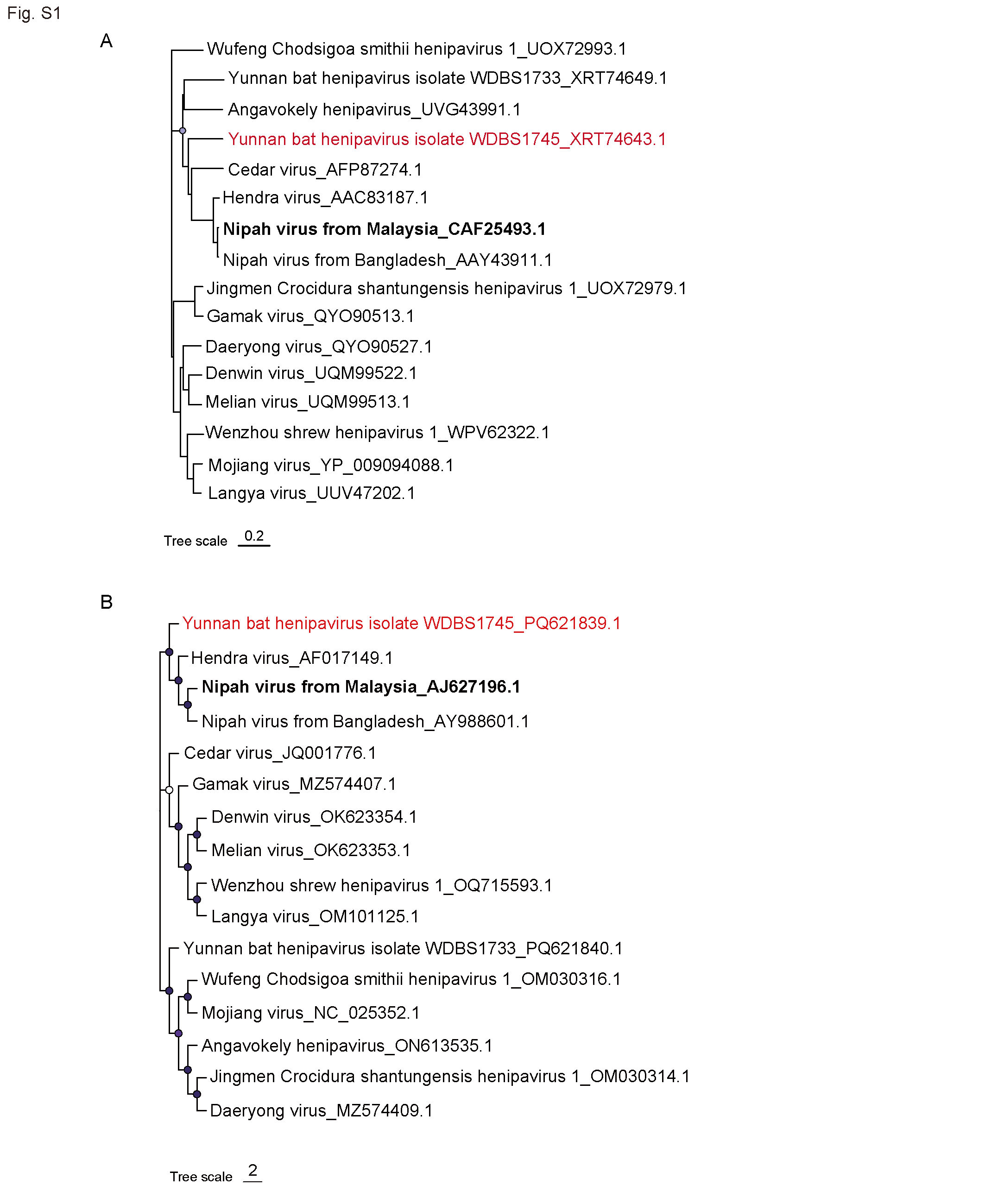

Supplement: S1 Fig — The phylogenetic tree was constructed based on the nucleocapsid protein sequences (A) and complete genome sequences (B) of selected paramyxoviruses. (TIF) [file pntd.0014557.s002.tif]

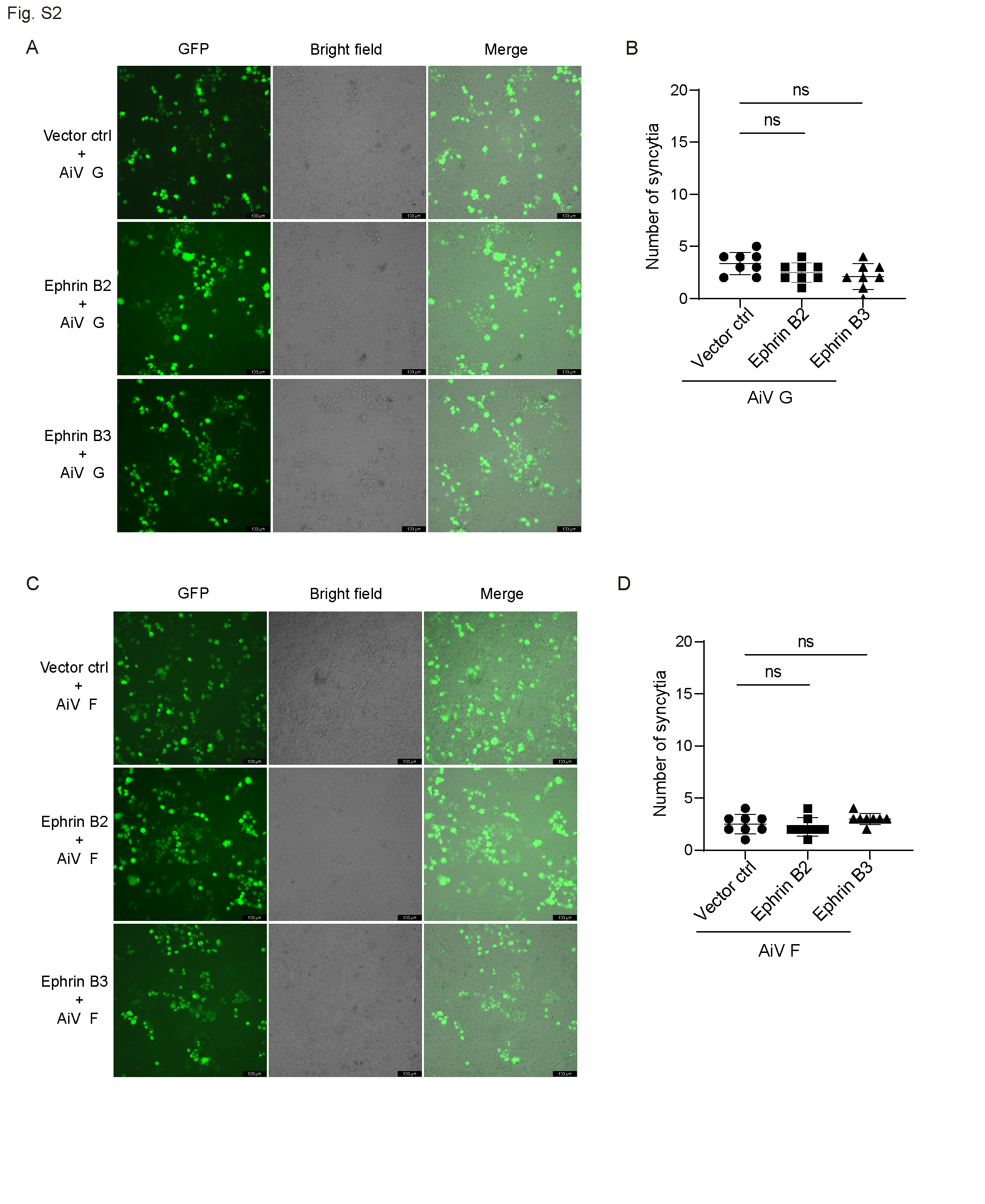

Supplement: S2 Fig — HEK293T cells transfected with AiV G (A and B), or F (C and D) were co-cultured with HEK293T cells expressing GFP together with EFNB2 or EFNB3, or the vector control. Cells were fixed and imaged by fluorescence microscopy to visualize the syncytia. Scale bar = 100 μm. (B, D) Quantification of syncytia from eight randomly selected microscopic fields per group. The experiments were conducted with three biological replicates. Data were analyzed by Student’s t-test (ns, not significant). (TIF) [file pntd.0014557.s003.tif]

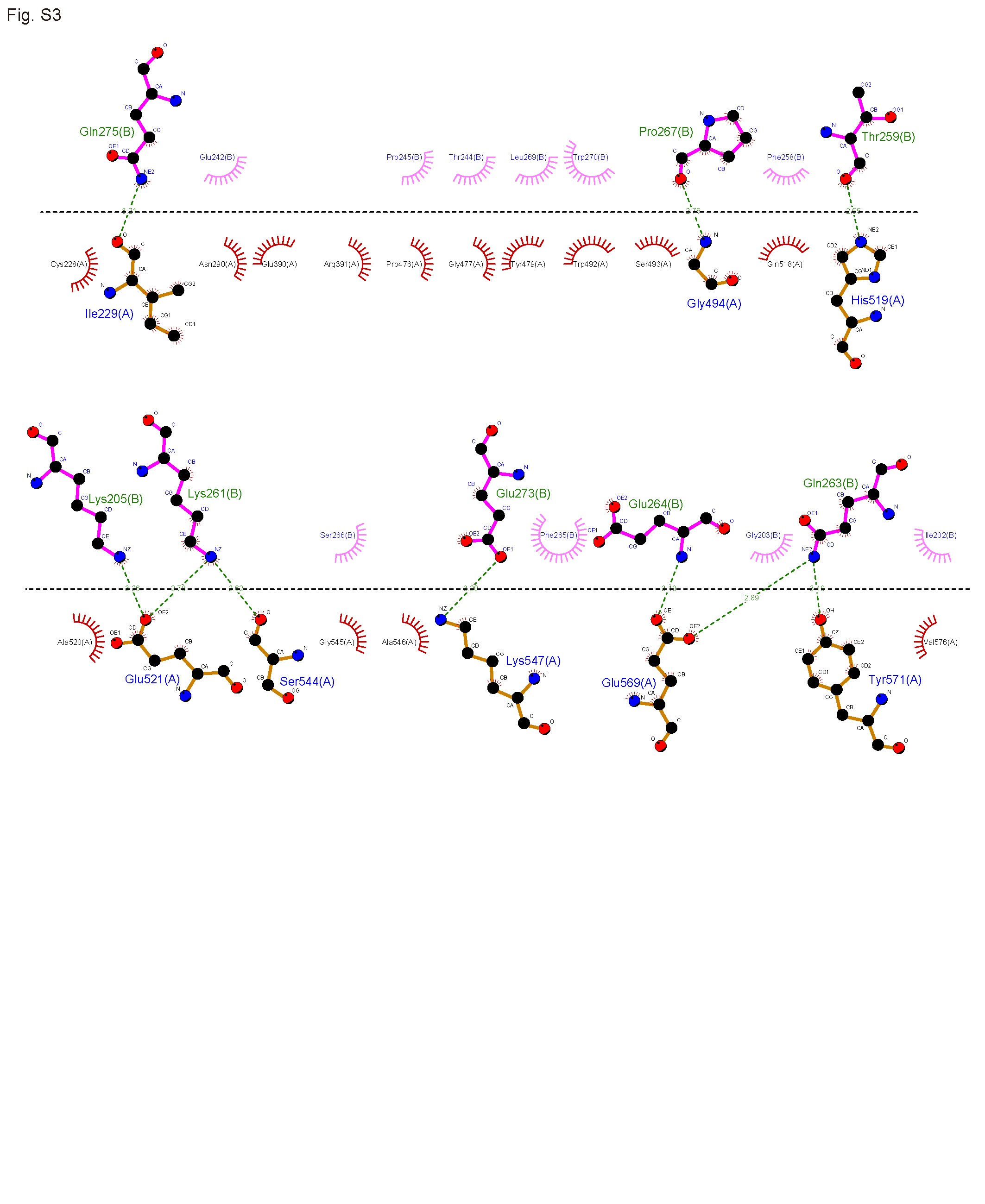

Supplement: S3 Fig — Hydrogen bonds are shown as green dashes; hydrophobic contacts as red/pink spoked arcs anchored to protein residues and directed toward ligand atom. The letter A in parentheses after an amino acid denotes AiV, and B in parentheses denotes EFNB2. (TIF) [file pntd.0014557.s004.tif]
